# Extending a Single Residue Switch for Abbreviating Catalysis in Plant *ent*-Kaurene Synthases

**DOI:** 10.3389/fpls.2016.01765

**Published:** 2016-11-22

**Authors:** Meirong Jia, Reuben J. Peters

**Affiliations:** Roy J. Carver Department of Biochemistry, Biophysics and Molecular Biology, Iowa State University, AmesIA, USA

**Keywords:** diterpenoids, diterpene synthases, product specificity, mutagenesis, metabolic engineering, carbocation, catalytic mechanism

## Abstract

Production of *ent*-kaurene as a precursor for important signaling molecules such as the gibberellins seems to have arisen early in plant evolution, with corresponding cyclase(s) present in all land plants (i.e., embryophyta). The relevant enzymes seem to represent fusion of the class II diterpene cyclase that produces the intermediate *ent*-copalyl diphosphate (*ent*-CPP) and the subsequently acting class I diterpene synthase that produces *ent*-kaurene, although the bifunctionality of the ancestral gene is only retained in certain early diverging plants, with gene duplication and sub-functionalization leading to distinct *ent*-CPP synthases and *ent*-kaurene synthases (KSs) generally observed. This evolutionary scenario implies that plant KSs should have conserved structural features uniquely required for production of *ent*-kaurene relative to related enzymes that have alternative function. Notably, substitution of threonine for a conserved isoleucine has been shown to “short-circuit” the complex bicyclization and rearrangement reaction catalyzed by KSs after initial cyclization, leading to predominant production of *ent*-pimaradiene, at least in KSs from angiosperms. Here this effect is shown to extend to KSs from earlier diverging plants (i.e., bryophytes), including a bifunctional/KS. In addition, attribution of the dramatic effect of this single residue “switch” on product outcome to electrostatic stabilization of the *ent*-pimarenyl carbocation intermediate formed upon initial cyclization by the hydroxyl introduced by threonine substitution has been called into question by the observation of similar effects from substitution of alanine. Here further mutational analysis and detailed product analysis is reported that supports the importance of electrostatic stabilization by a hydroxyl or water.

## Introduction

**SCHEME 1:**
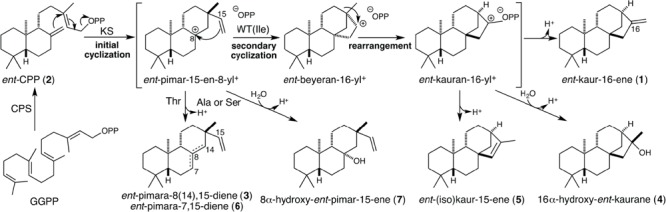
**Cyclization mechanism catalyzed by KSs and the Ile mutants investigated here.** These KS catalyzed reactions are initiated by ionization of bicyclic *ent*-CPP (**2**), generated from the acyclic precursor GGPP by CPS, with initial cyclization to *ent*-pimar-15-en-8-yl^+^. In Ile→Thr mutants this intermediate is directly deprotonated, affording largely *ent*-pimara-8(14),15-diene (**4**) with small amounts of *ent*-pimara-7,15-diene (**6**), while Ile→Ala or Ser mutants also produce significant amounts of 8α-hydroxy-*ent*-pimara-15-ene (**7**), generated by addition of water prior to deprotonation. In wild-type (WT) KSs secondary cyclization occurs, followed by ring rearrangement, with deprotonation of the neighboring methyl group yielding *ent*-kaur-16-ene (**1**). Some of the wild-type KS(L)s investigated here produce either *ent*-isokaur-15-ene (**5**) (i.e., OsKSL5i), or a mixture of **1** and 16α-hydroxy-*ent*-kaurane (**4**) (i.e., PpCPS/KS), with the latter generated by addition of water prior to deprotonation. Numbers correspond to the compound numbering defined in text.

All embryophyta produce the diterpene *ent*-kaur-16-ene (**1**). In vascular plants (i.e., tracheophytes) **1** serves as an intermediate in biosynthesis of the gibberellin phytohormones (Hedden and Thomas, 2012). Even in earlier diverging bryophytes **1** seems to serve as a precursor to an as yet undefined signaling molecule (Anterola et al., 2009; Hayashi et al., 2010; Miyazaki et al., 2014, 2015). Production of **1** from the general diterpenoid precursor (*E,E,E*)-geranylgeranyl diphosphate (GGPP) proceeds via two distinct bicyclization reactions (Peters, 2010). The first is catalyzed by copalyl diphosphate synthases (CPSs) that are representative of class II diterpene cyclases and produce *ent*-CPP (**2**). **2** is then subsequently further cyclized and rearranged by KSs that are representative of class I (di)terpene synthases (Zi et al., 2014). While the reactions catalyzed by CPSs and KSs are mechanistically distinct and carried out in distinct active sites, in plants these enzymes seem to be derived from fusion of the genes for each enzyme – i.e., *CPS* and *KS* – potentially from bacteria (Morrone et al., 2009; Gao et al., 2012). However, the presumably bifunctional nature of the initially resulting enzyme appears to have been retained only in certain bryophytes (Hayashi et al., 2006; Kawaide et al., 2011; Kumar et al., 2016). The production of **1** in most plants appears to depend on separate CPS and KS that arose from duplication and sub-functionalization of the ancestral fused bifunctional enzyme, with retention of the original multi-domain structure (Zi et al., 2014). In turn, the CPS and KS required for the production of signaling molecules derived from **1**, such as the gibberellins, seem to have given rise to closely related enzymes that mediate more specialized labdane-related diterpenoid metabolism via gene duplication and neo-functionalization (Zi et al., 2014). These enzymes then catalyze similar reactions, but yield different products. For example, many angiosperms contain small families of KS-like diterpene synthases (KSLs) that retain the multi-domain structure of KSs, but do not produce **1**. These catalyze ionization of the allylic diphosphate ester bond that characterizes class I terpene synthases, and may even still react with **2**. However, these enzymes then mediate distinct product outcome by either abbreviation of the KS reaction, or by mediating formation of a distinct series of carbocationic intermediates. These KSLs terminate catalysis via deprotonation, either directly or following the addition of water, leading to a variety of products (see **Scheme 1** for examples).

The evolutionary scenario presented above implies that the ancestral enzyme produced *ent*-kaurene, with conservation of this function in all extant KSs. However, such conservation of function versus parallel evolution from related but functionally divergent ancestors is difficult to discern from phylogenetic analyses given the extended timescale over which land plants have evolved (Kumar et al., 2016). For example, while KSs contain highly conserved DDxxD and NDxx(G/S/T)xxxE amino acid sequence motifs, these are found in class I terpene synthases more generally. Specifically, because they are involved in binding magnesium divalent ion co-factors required for the initiating ionization of the allylic diphosphate ester bond (Christianson, 2006). On the other hand, KSs have been reported to contain a conserved isoleucine that plays a surprisingly critical role in promoting the complex bicyclization and rearrangement reaction catalyzed by these enzymes (**Scheme 1**). In particular, substitution of threonine for this Ile leads to an abbreviated reaction, only cyclization of the *ent*-CPP substrate to *ent*-pimara-8(14),15-diene (**3**), representing deprotonation of the *ent*-pimar-15-en-8-yl carbocation formed by initial cyclization (Xu et al., 2007b). This effect has been interpreted as resulting from electrostatic stabilization of this carbocationic intermediate by the hydroxyl of the introduced Thr side-chain, rather than the hydroxyl group acting as a general base. The presence of the inert Ile hydrocarbon side-chain then allows carbocation migration toward the diphosphate anion co-product via further cyclization and ring rearrangement prior to terminating deprotonation (Zhou and Peters, 2011). However, conservation of this Ile and the effect of Thr substitution was originally only reported for KSs from angiosperms (Xu et al., 2007b). This Ile is conserved in a KS from gymnosperms (Keeling et al., 2010), with a similar effect on product outcome observed upon substitution with alanine as well (Zerbe et al., 2012). Thus, this Ile seems to be conserved in KSs throughout seed plants (i.e., spermatophyta). However, the identity and importance of this residue has not yet been investigated in KSs from even earlier diverging plants. Moreover, the similar effect of Thr or Ala substitution on product outcome also leaves some question as to the mechanistic role of the residue at this position given that Ala would be expected to be as inert as Ile. Here the conservation of this Ile and effect of Thr substitution is extended to KSs from the earliest diverging plants (i.e., bryophytes), including an example of a bifunctional CPS/KS, along with additional mutational analysis of this critical residue that resolve its role in the enzymatically catalyzed reaction.

## Materials and Methods

### General

Unless otherwise noted, chemicals were purchased from Fisher Scientific and molecular biology reagents from Invitrogen. Sequence alignments were performed with CLC sequence viewer 6.9.1 using default parameters.

### Mutant Construction

The enzymes investigated here are pseudomature constructs suitable for recombinant expression in *Escherichia coli*, as previously described for MpKS (Kumar et al., 2016), PpCPS/KS (Hayashi et al., 2006), OsKSL5i (Xu et al., 2007a), and AtKS (Yamaguchi et al., 1998). Site-directed mutants were constructed by whole-plasmid PCR amplification of the relevant pENTR/SD/d-TOPO constructs using the primers described in Supplementary Table S1, and AccuPrime^TM^ Pfx DNA Polymerase. All mutants were verified by complete gene sequencing, and then transferred via directional recombination to the T7-based N-terminal GST fusion expression vector pDEST15.

### Enzymatic Analyses

To determine catalyzed product outcome, each pDEST15 based construct was co-transformed with a previously described pGG*e*C vector containing both a GGPP synthase and *ent*-CPP synthase (Cyr et al., 2007), along with a previously reported pIRS plasmid that increases metabolic flux toward terpenoids (Morrone et al., 2010), into the OverExpress C41 strain of *Escherichia coli* (Lucigen). The resulting recombinant strains were cultured in 50 mL TB medium (pH = 7.0), with appropriate antibiotics, in 250 mL Erlenmeyer flasks. These cultures were first grown by shaking at 37°C to mid-log phase (OD_600_∼0.7), then the temperature dropped to 16°C for 0.5 h prior to induction with 1 mM isopropylthiogalactoside (IPTG) and supplementation with 40 mM pyruvate and 1 mM MgCl_2_. The induced cultures were further grown for an additional 72 h before extraction with an equal volume of hexanes, with the organic phase then separated, and concentrated under N_2_ when necessary.

### Product Analyses

Gas chromatography with mass spectral detection (GC-MS) was carried on a Varian 3900 GC with a Saturn 2100T ion trap mass spectrometer in electron ionization (70 eV) mode, using an Agilent HP-5MS column (Agilent, 19091S-433) with 1.2 mL/min helium flow rate. Samples (1 μL) were injected in splitless mode by an 8400 autosampler with the injection port set at 250°C. The following temperature program was used: the oven temperature initially started at 50°C, which was maintained for 3 min, and then increased at a rate of 15°C/min to 300°C, where it was held for another 3 min. Mass spectrum was recorded by mass-to-charge ratio (*m/z*) values in a range from 90 to 650, starting from 13 min after sample injection until the end of the run. Enzymatic products were identified by comparison of retention time and mass spectra to those of authentic standards.

## Results

### Extending the Conservation of a Key Residue in Plant KSs

**FIGURE 1:**
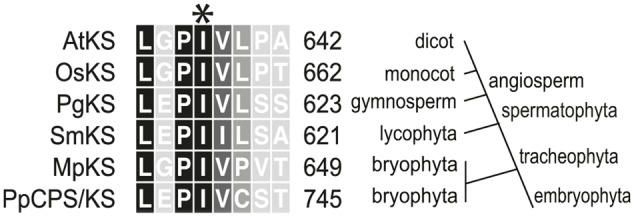
**Partial sequence alignment of representative kaurene synthases spanning plant evolution.** The highly conserved Ile probed here is indicated by an asterisk (^∗^). Residues are numbered as in the full length enzymes. The phylogenetic terms on the right apply to the divisions branching above that point. The aligned sequences are named as follows (NCBI protein database accession): AtKS, *Arabidopsis thaliana* KS (AAC39443); OsKS, *Oryza sativa* KS (BAE72099); PgKS, *Picea glauca* KS (ADB55711); SmKS, *Selaginella moellendorffii* KS (BAP19110); PpCPS/KS, *Physcomitrella patens* KS (BAF61135); MpKS, *Marchantia polymorpha* KS (OAE22677).

**FIGURE 2:**
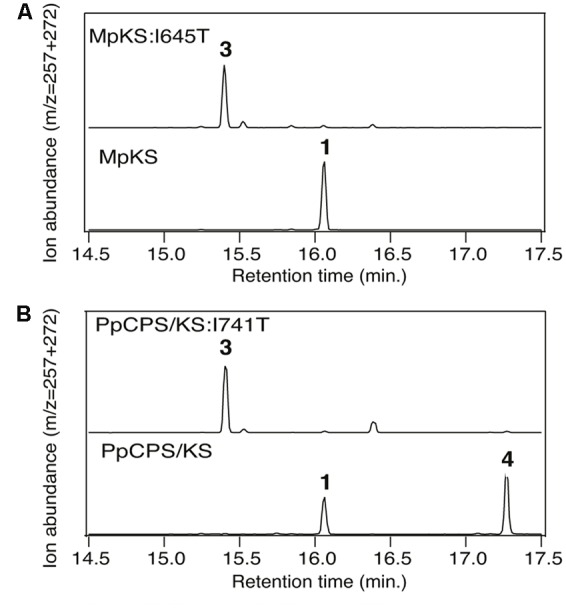
**Effect of Ile-to-Thr mutation on (hydroxy)kaurene synthase product outcome.** Chromatograms from GC–MS analysis of the indicated diterpene synthases (wild-type or indicated mutant). **(A)** MpKS. **(B)** PpCPS/KS. Numbers correspond to the compound numbering defined in the text (i.e., **1**, *ent*-kaurene; **3**, *ent*-pimara-8(14),15-diene; **4**, 16α-hydroxy-*ent*-kaurane). Enzymatic products were identified by comparison of both retention time and mass spectra to authentic standards (see Supplementary Figure S1).

Intriguingly, a recently identified monofunctional KS from the liverwort *Marchantia polymorpha* (MpKS) (Kumar et al., 2016) was found to contain an Ile at the key position previously identified in spermatophyta KSs (**Figure 1**). Moreover, this Ile is further conserved in the only characterized KS from a lycophyte (i.e., *Selaginella moellendorffii*, SmKS) (Shimane et al., 2014), as well as the bifunctional CPS/KSs identified from other bryophytes (Hayashi et al., 2006; Kawaide et al., 2011). This includes that from the moss *Physcomitrella patens* (PpCPS/KS), which produces a mixture of *ent*-kaurene and 16α-hydroxy-*ent*-kaurane (**4**) (Hayashi et al., 2006). To determine if this Ile was similarly important in these phylogenetically and functionally disparate KSs, Thr substitution mutants were constructed in both MpKS (I645T) and PpCPS/KS (I741T). The effect of these mutations on product outcome was investigated by recombinant expression in *Escherichia coli*. Specifically, by use of a previously developed modular metabolic engineering system that enables bacterial co-production of the appropriate substrate (Cyr et al., 2007), here *ent*-CPP (**2**), along with the KSs (either wild-type or mutant). Strikingly, both MpKS:I645T and PpCPS/KS:I741T produced almost entirely *ent*-pimara-8(14),15-diene (**3**). This contrasts with the almost exclusive production of *ent*-kaurene (**1**) by the wild-type MpKS, and mixture of **1** and **4** produced by the wild-type PpCPS/KS (**Figure 2**). These results then extend the conserved functional importance of this Ile to KSs across the embryophyta phylogeny.

### Extending Understanding of the Role of the Key Ile Residue in KS Catalysis

**FIGURE 3:**
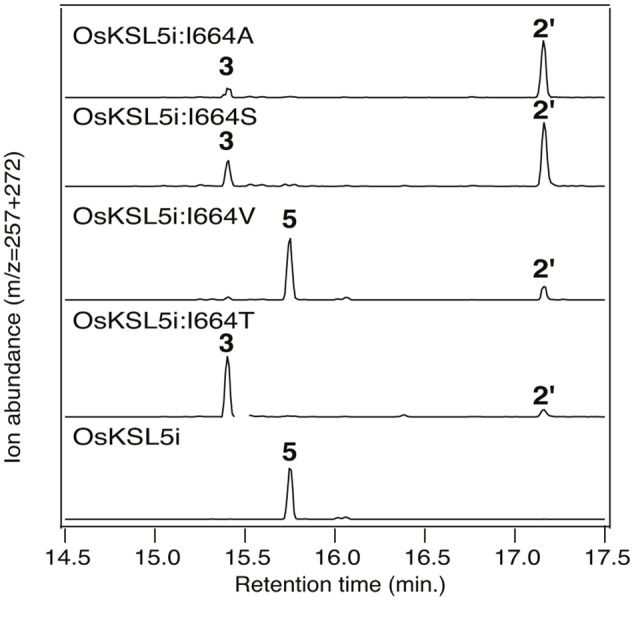
**Effect of substitutions for I664 on OsKSL5i product outcome.** Chromatograms from GC–MS analysis of the indicated diterpene synthases (wild-type or indicated mutant). Numbers correspond to the compound numbering defined in the text (i.e., **3**, *ent*-pimara-8(14),15-diene; **5**, *ent*-isokaurene), with **2′** corresponding to *ent*-copalol, the dephosphorylated derivative of *ent*-CPP (**2**). Enzymatic products were identified by comparison of both retention time and mass spectra to authentic standards (see Supplementary Figure S1).

It has been suggested that the effect of Thr substitution for the key Ile is due to electrostatic stabilization of the pimar-15-en-8-yl carbocation immediately formed by initial cyclization, enabling deprotonation (Zhou and Peters, 2011). However, similar effects on product outcome have been reported for Ala substitution for the corresponding Ile in the KS from the gymnosperm *Picea glauca* (Zerbe et al., 2012). Given the lack of a hydroxyl group in Ala, these results leave the original mechanistic interpretation in question. This discrepancy was first addressed by constructing the equivalent Ala substitution mutant for the *ent*-isokaur-15-ene synthase from rice (OsKSL5i) (Xu et al., 2007a), in which the effect of Thr substitution was originally discovered (Xu et al., 2007b). Notably, this OsKSL5i:I664A mutant produced almost exclusively *ent*-pimara-8(14),15-diene (**3**), albeit only in small quantities. To further probe the basis for this effect on product outcome, additional mutants were constructed, substituting serine or valine at this position in OsKSL5i. Interestingly, these showed opposite effects. The OsKSL5i:I664S mutant produced largely **3**, much like the previously described OsKSL5i:I664T mutant (Xu et al., 2007b). By contrast, the OsKSL5i:I664V mutant predominantly produced *ent*-(iso)kaur-15-ene (**5**), much like the wild-type enzyme (**Figure 3**). Given the minimal difference between the volume of Thr and Val side chains, it seems unlikely that these results are explicable by steric effects. An alternative hypothesis is that substitution by Ala leaves space that is occupied by a water molecule, providing similar electrostatic stabilization of the *ent*-pimar-15-en-8-yl^+^ intermediate as substitution by the hydroxyl containing Thr and Ser side chains. However, it seems puzzling that such a water is not observed to add to this carbocation, which would yield a hydroxylated product.

**FIGURE 4:**
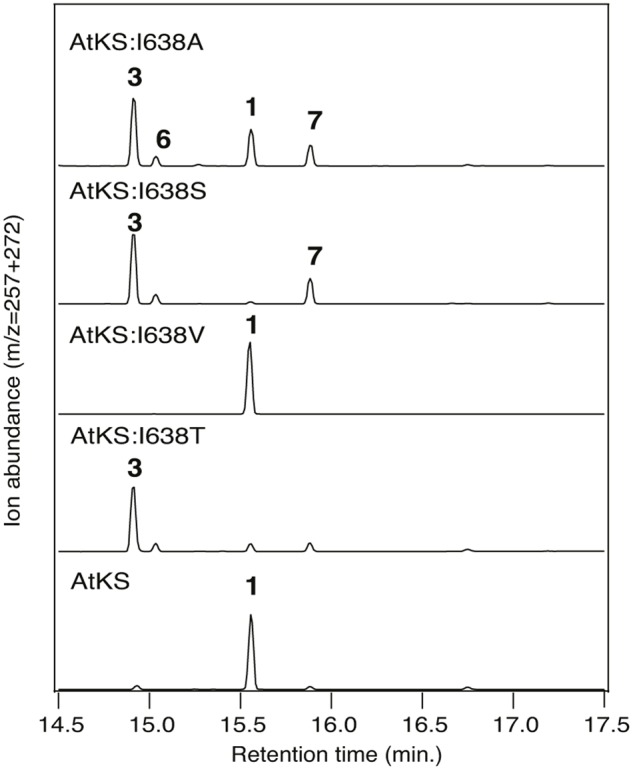
**Effect of various substitutions for I638 on AtKS product outcome.** Chromatograms from GC–MS analysis of the indicated diterpene synthases (wild-type or indicated mutant). Numbers correspond to the compound numbering defined in the text (i.e., **1**, *ent*-kaurene; **3**, *ent*-pimara-8(14),15-diene; **6**, *ent*-pimara-7,15-diene; **7**, 8α-hydroxy-*ent*-pimar-15-ene). Enzymatic products were identified by comparison of both retention time and mass spectra to authentic standards (see Supplementary Figure S2).

Intriguingly, although not observed with OsKSL5i:I664A, the equivalent PgKS:I619A mutant was reported to make at least two unidentified minor products, although it was not reported if either was hydroxylated (Zerbe et al., 2012). Given that all of the OsKSL5i mutants exhibited decreased yield in the modular metabolic engineering system, as evidenced by presence of significant amounts of *ent*-copalol (**2′**) derived from dephosphorylation of **2** (presumably by endogenous phosphatases), it seemed worth investigating a more robust KS. Previous work indicated that Thr substitution for the key Ile in the KS from *Arabidopsis thaliana* (AtKS) retains good activity. In particular, no **2′** was observed with this AtKS:I638T mutant (Xu et al., 2007b). Accordingly, an equivalent set of additional substitutions (i.e., Ala, Ser, or Val) at this position were constructed in AtKS. While the overall impact of these mutations on product outcome in the modular metabolic engineering system were analogous to those observed with OsKSL5i, additional products were observed (**Figure 4**). In particular, while the AtKS:I638V mutant produced just *ent*-kaurene (**1**), the AtKS:I638A and AtKS:I638S mutants produced a mixture of four products. Beyond the predominant production of *ent*-pimara-8(14),15-diene (**3**), these also produced small amounts of the double bond isomer *ent*-pimara-7,15-diene (**6**) and variable amounts of **1**, as well as substantial amounts of a hydroxylated product. This diterpene alcohol was shown to be 8α-hydroxy-*ent*-pimar-15-ene (**7**) by comparison to an authentic standard. **7** almost certainly results from stereospecific addition of water to the *ent*-pimar-15-en-8-yl^+^ intermediate, with subsequent deprotonation. Upon incorporation into the modular metabolic engineering system with increased flux, it was further found that the AtKS:I638T mutant also exhibited a similar mixture of the same four products, albeit with less of the hydroxylated **7** observed (**Figure 4**). This provides a strong contrast to the approximately isosteric AtKS:I638V mutant, and indicates that the hydroxyl group of the Thr side chain enables occasional addition of water.

## Discussion

**SCHEME 2:**
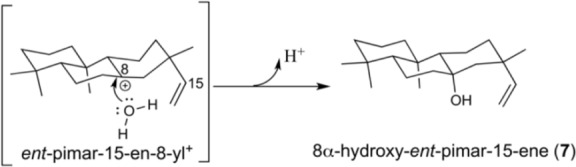
**Schematic depicting the water bound in the active site of the AtKS:I638S/A mutants, and its addition to yield 7**.

The results reported here not only demonstrate conservation of the unique importance of a key Ile in the complex cyclization and rearrangement reaction catalyzed by plant KSs throughout the embryophyta, but also further extend our understanding of the underlying mechanism. For example, the stereospecific addition of water to generate the 8α-hydroxy-*ent*-pimar-15-ene product (**7**) observed upon substitution of the relevant I638 in AtKS indicates the orientation of this residue relative to the *ent*-pimar-15-en-8-yl^+^ intermediate in the catalyzed reaction. Specifically, in order for this to occur, the water must attack from ‘underneath’ the ring structure (**Scheme 2**). This presumably further defines the relative positioning of the key Ile residue, as it is changes of this amino acid side chain that enable such access.

Although the observed effects might suggest the hypothesis that these substitutions enable water to bind and act as a general base that directly deprotonates the *ent*-pimar-15-en-8-yl^+^ intermediate, this seems somewhat unlikely given previous results reported for class II diterpene cyclases that also catalyze reactions with similar carbocationic intermediates. In particular, while there is strong evidence that a specific water acts as the general base in the *ent*-CPP synthase from *A. thaliana*, this water is tightly constrained by several interactions with the enzyme. This includes the side chains of a histidine and asparagine, substitution of either of which with Ala is sufficient to lead to predominant formation of a hydroxylated product (Potter et al., 2014). Moreover, simply opening up space to create a polar ‘pocket’ at the site of the catalytic base in the class II diterpene cyclase active site of the abietadiene synthase from *Abies grandis* (i.e., either substitution of Ala or aspartate for histidine, or phenylalanine for the hydrogen-bonded tyrosine) similarly leads to specific addition of water and predominant production of hydroxylated products (Criswell et al., 2012; Mafu et al., 2015). In both cases, this contrasts with the occasional addition of water and relatively small amounts of hydroxylated product observed here (**Figure 4**). Accordingly, it seems unlikely that introducing a single hydroxyl group would be sufficient to both bind and activate a water to largely function as a general base, particularly without adding to the carbocation more often than is observed. Thus, although such a direct mechanism cannot be entirely ruled out, it seems most likely that the observed effects are due to the electrostatic effect of the introduced hydroxyl on stabilization of the *ent*-pimar-15-en-8-yl^+^ for deprotonation. The water bound in the cavity created by substitution of Ala for this Ile would then similarly enable the effects on product outcome observed here with such I→A mutants of KSs (**Figures 3** and **4**). In this mechanism the general base is most likely the pyrophosphate anion co-product, as previously suggested for class I terpene synthases in general (Pemberton and Christianson, 2016).

Regardless of the exact role of this key Ile, the conservation of this residue across all known embryophyta KSs is consistent with the hypothesis that the earliest plant terpene synthase was a bifunctional CPS/KS (e.g., much like the PpCPS/KS studied here). This hypothesis is further reinforced by the observation that the key Ile from plant KSs is not found in the functionally analogous enzymes that have been identified in microbes (Morrone et al., 2009). In particular, this Ile is not conserved in the bifunctional CPS/KS found in certain fungi (Bomke and Tudzynski, 2009), which otherwise nominally resemble those found in plants (Peters, 2013). Nor is this Ile conserved in the known single-domain monofunctional KSs in bacteria, where such activity appears to have arisen via parallel evolution in two separate contexts (Morrone et al., 2009; Smanski et al., 2011; Hershey et al., 2014; Lu et al., 2015). The widespread functional conservation of a key Ile shown here indicates an early appearance of KSs in plant evolution, which is consistent with the importance of *ent*-kaurene derived molecules in all embryophyta, even the bryophytes that do not produce gibberellins (Hirano et al., 2007; Yasumura et al., 2007). Such early origins for KSs also is consistent with the previously suggested staggered evolution of gibberellin biosynthesis in which modification of the *ent*-kaurane backbone into gibberellins only arose after divergence of the bryophytes from tracheophytes (Zi et al., 2014). Moreover, the catalytic plasticity of these enzymes demonstrated here is further consistent with the hypothesis that the KSs gave rise to the plant family of classic (class I) terpene synthases more generally, the importance of which in the diversification of plant metabolism is highlighted by their presence as moderately large gene families in all tracheophytes (Chen et al., 2011).

## Author Contributions

MJ designed and conducted the experiments. MJ and RP jointly conceived of the project, interpreted the data, and wrote the paper.

## Conflict of Interest Statement

The authors declare that the research was conducted in the absence of any commercial or financial relationships that could be construed as a potential conflict of interest.

## References

[B1] Anterola A. Shanle E. Mansouri K. Schuette S. Renzaglia K. (2009). Gibberellin precursor is involved in spore germination in the moss *Physcomitrella patens*. *Planta* 229 1003–1007. 10.1007/s00425-008-0875-1 19112579

[B2] Bomke C. Tudzynski B. (2009). Diversity, regulation, and evolution of the gibberellin biosynthetic pathway in fungi compared to plants and bacteria. *Phytochemistry* 70 1876–1893. 10.1016/j.phytochem.2009.05.020 19560174

[B3] Chen F. Tholl D. Bohlmann J. Pichersky E. (2011). The family of terpene synthases in plants: a mid-size family of genes for specialized metabolism that is highly diversified throughout the kingdom. *Plant J.* 66 212–229. 10.1111/j.1365-313X.2011.04520.x 21443633

[B4] Christianson D. W. (2006). Structural biology and chemistry of the terpenoid cyclases. *Chem. Rev.* 106 3412–3442. 10.1021/cr050286w 16895335

[B5] Criswell J. Potter K. Shephard F. Beale M. B. Peters R. J. (2012). A single residue change leads to a hydroxylated product from the class II diterpene cyclization catalyzed by abietadiene synthase. *Org. Lett.* 14 5828–5831. 10.1021/ol3026022 23167845 PMC3518578

[B6] Cyr A. Wilderman P. R. Determan M. Peters R. J. (2007). A modular approach for facile biosynthesis of labdane-related diterpenes. *J. Am. Chem. Soc.* 129 6684–6685. 10.1021/ja071158n 17480080 PMC2518946

[B7] Gao Y. Honzatko R. B. Peters R. J. (2012). Terpenoid synthase structures: a so far incomplete view of complex catalysis. *Nat. Prod. Rep.* 29 1153–1175. 10.1039/c2np20059g 22907771 PMC3448952

[B8] Hayashi K. Horie K. Hiwatashi Y. Kawaide H. Yamaguchi S. Hanada A. (2010). Endogenous diterpenes derived from *ent*-kaurene, a common gibberellin precursor, regulate protonema differentiation of the moss *Physcomitrella patens*. *Plant Physiol.* 153 1085–1097. 10.1104/pp.110.157909 20488896 PMC2899919

[B9] Hayashi K. Kawaide H. Notomi M. Sakigi Y. Matsuo A. Nozaki H. (2006). Identification and functional analysis of bifunctional ent-kaurene synthase from the moss *Physcomitrella patens*. *FEBS Lett.* 580 6175–6181. 10.1016/j.febslet.2006.10.018 17064690

[B10] Hedden P. Thomas S. G. (2012). Gibberellin biosynthesis and its regulation. *Biochem. J.* 444 11–25. 10.1042/BJ20120245 22533671

[B11] Hershey D. M. Lu X. Zi J. Peters R. J. (2014). Functional conservation of the capacity for ent-kaurene biosynthesis and an associated operon in certain rhizobia. *J. Bact.* 196 100–106. 10.1128/JB.01031-13 24142247 PMC3911121

[B12] Hirano K. Nakajima M. Asano K. Nishiyama T. Sakakibara H. Kojima M. (2007). The GID1-mediated gibberellin perception mechanism is conserved in the Lycophyte *Selaginella moellendorffii* but not in the Bryophyte *Physcomitrella patens*. *Plant Cell* 19 3058–3079. 10.1105/tpc.107.051524 17965273 PMC2174699

[B13] Kawaide H. Hayashi K. Kawanabe R. Sakigi Y. Matsuo A. Natsume M. (2011). Identification of the single amino acid involved in quenching the *ent*-kauranyl cation by a water molecule in ent-kaurene synthase of *Physcomitrella patens*. *FEBS J.* 278 123–133. 10.1111/j.1742-4658.2010.07938.x 21122070

[B14] Keeling C. I. Dullat H. K. Yuen M. Ralph S. G. Jancsik S. Bohlmann J. (2010). Identification and functional characterization of monofunctional *ent*-copalyl diphosphate and *ent*-kaurene synthases in white spruce reveal different patterns for diterpene synthase evolution for primary and secondary metabolism in gymnosperms. *Plant Physiol.* 152 1197–1208. 10.1104/pp.109.151456 20044448 PMC2832265

[B15] Kumar S. Kempinski C. Zhuang X. Norris A. Mafu S. Zi J. (2016). Molecular Diversity of terpene synthases in the liverwort *Marchantia polymorpha*. *Plant Cell* 10.1105/tpc.16.00062 [Epub ahead of print]. PMC5134972 27650333

[B16] Lu X. Hershey D. M. Wang L. Bogdanove A. J. Peters R. J. (2015). An *ent*-kaurene derived diterpenoid virulence factor from *Xanthomonas oryzae* pv. oryzicola. *New Phytol.* 406 295–302. 10.1111/nph.13187 25406717

[B17] Mafu S. Potter K. C. Hillwig M. L. Schulte S. Criswell J. Peters R. J. (2015). Efficient heterocyclisation by (di)terpene synthases. *Chem. Commun. (Camb)* 51 13485–13487. 10.1039/c5cc05754j 26214384 PMC4543578

[B18] Miyazaki S. Nakajima M. Kawaide H. (2015). Hormonal diterpenoids derived from ent-kaurenoic acid are involved in the blue-light avoidance response of *Physcomitrella patens*. *Plant Signal. Behav.* 10:e989046 10.4161/15592324.2014.989046 PMC4622475 25751581

[B19] Miyazaki S. Toyoshima H. Natsume M. Nakajima M. Kawaide H. (2014). Blue-light irradiation up-regulates the ent-kaurene synthase gene and affects the avoidance response of protonemal growth in *Physcomitrella patens*. *Planta* 240 117–124. 10.1007/s00425-014-2068-4 24715198

[B20] Morrone D. Chambers J. Lowry L. Kim G. Anterola A. Bender K. (2009). Gibberellin biosynthesis in bacteria: separate *ent*-copalyl diphosphate and *ent*-kaurene synthases in *Bradyrhizobium japonicum*. *FEBS Lett.* 583 475–480. 10.1016/j.febslet.2008.12.052 19121310

[B21] Morrone D. Lowry L. Determan M. K. Hershey D. M. Xu M. Peters R. J. (2010). Increasing diterpene yield with a modular metabolic engineering system in *E. coli*: comparison of MEV and MEP isoprenoid precursor pathway engineering. *Appl. Microbiol. Biotechnol.* 85 1893–1906. 10.1007/s00253-009-2219-x 19777230 PMC2811251

[B22] Pemberton T. A. Christianson D. W. (2016). General base-general acid catalysis by terpenoid cyclases. *J. Antibiotics* 69 486–493. 10.1038/ja.2016.39 27072285 PMC4963284

[B23] Peters R. J. (2010). Two rings in them all: the labdane-related diterpenoids. *Nat. Prod. Rep.* 27 1521–1530. 10.1039/c0np00019a 20890488 PMC3766046

[B24] Peters R. J. (2013). “Gibberellin phytohormone metabolism,” in *Isoprenoid Synthesis in Plants and Microorganisms: New Concepts and Experimental Approaches* eds Bach T. Rohmer M. (New York: Springer) 233–249.

[B25] Potter K. Criswell J. Peters R. J. (2014). Novel product chemistry from mechanistic analysis of *ent*-copalyl diphosphate synthases from plant hormone biosynthesis. *Angew. Chem. Int. Ed.* 53 7198–7202. 10.1002/anie.201402911 PMC4113509 24862907

[B26] Shimane M. Ueno Y. Morisaki K. Oogami S. Natsume M. Hayashi K. (2014). Molecular evolution of the substrate specificity of ent-kaurene synthases to adapt to gibberellin biosynthesis in land plants. *Biochem. J.* 462 539–546. 10.1042/BJ20140134 24983886

[B27] Smanski M. J. Yu Z. Casper J. Lin S. Peterson R. M. Chen Y. (2011). Dedicated ent-kaurene and ent-atiserene synthases for platensimycin and platencin biosynthesis. *Proc. Natl. Acad. Sci. U.S.A.* 108 13498–13503. 10.1073/pnas.1106919108 21825154 PMC3158216

[B28] Xu M. Wilderman P. R. Morrone D. Xu J. Roy A. Margis-Pinheiro M. (2007a). Functional characterization of the rice kaurene synthase-like gene family. *Phytochemistry* 68 312–326. 10.1016/j.phytochem.2006.10.016 17141283

[B29] Xu M. Wilderman P. R. Peters R. J. (2007b). Following evolution’s lead to a single residue switch for diterpene synthase product outcome. *Proc. Natl. Acad. Sci. U.S.A.* 104 7397–7401. 10.1073/pnas.0611454104 17456599 PMC1855280

[B30] Yamaguchi S. Sun T. Kawaide H. Kamiya Y. (1998). The GA2 locus of *Arabidopsis thaliana* encodes *ent*-kaurene synthase of gibberellin biosynthesis. *Plant Physiol.* 116 1271–1278. 10.1104/pp.116.4.1271 9536043 PMC35033

[B31] Yasumura Y. Crumpton-Taylor M. Fuentes S. Harberd N. P. (2007). Step-by-step acquisition of the gibberellin-DELLA growth-regulatory mechanism during land-plant evolution. *Curr. Biol.* 17 1225–1230. 10.1016/j.cub.2007.06.037 17627823

[B32] Zerbe P. Chiang A. Bohlmann J. (2012). Mutational analysis of white spruce (*Picea glauca*) ent-kaurene synthase (PgKS) reveals common and distinct mechanisms of conifer diterpene synthases of general and specialized metabolism. *Phytochemistry* 74 30–39. 10.1016/j.phytochem.2011.11.004 22177479

[B33] Zhou K. Peters R. J. (2011). Electrostatic effects on (di)terpene synthase product outcome. *Chem. Commun.* 47 4074–4080. 10.1039/c0cc02960b PMC3718459 21305070

[B34] Zi J. Mafu S. Peters R. J. (2014). To gibberellins and beyond! surveying the evolution of (di)terpenoid metabolism. *Annu. Rev. Plant Biol.* 65 259–286. 10.1146/annurev-arplant-050213-035705 24471837 PMC4118669

