# Supplementary material for: Extending a Single Residue Switch for Abbreviating Catalysis in Plant ent-Kaurene Synthases
Source: Front Plant Sci. 2016 Nov 22;7:1765. doi: 10.3389/fpls.2016.01765 (PMC5118566; doi:10.3389/fpls.2016.01765)
Supplement: Supplementary file 1 [file Data_Sheet_1.pdf]

Supplementary Material for:

Extending a Single Residue Switch for Abbreviating Catalysis in Plant *ent*-Kaurene Synthases

Meirong Jia and Reuben J. Peters*

Roy J. Carver Department of Biochemistry, Biophysics & Molecular Biology, Iowa State University, Ames, IA 50011, USA

*** Correspondence:**

Reuben J. Peters

rjpeters@iastate.edu

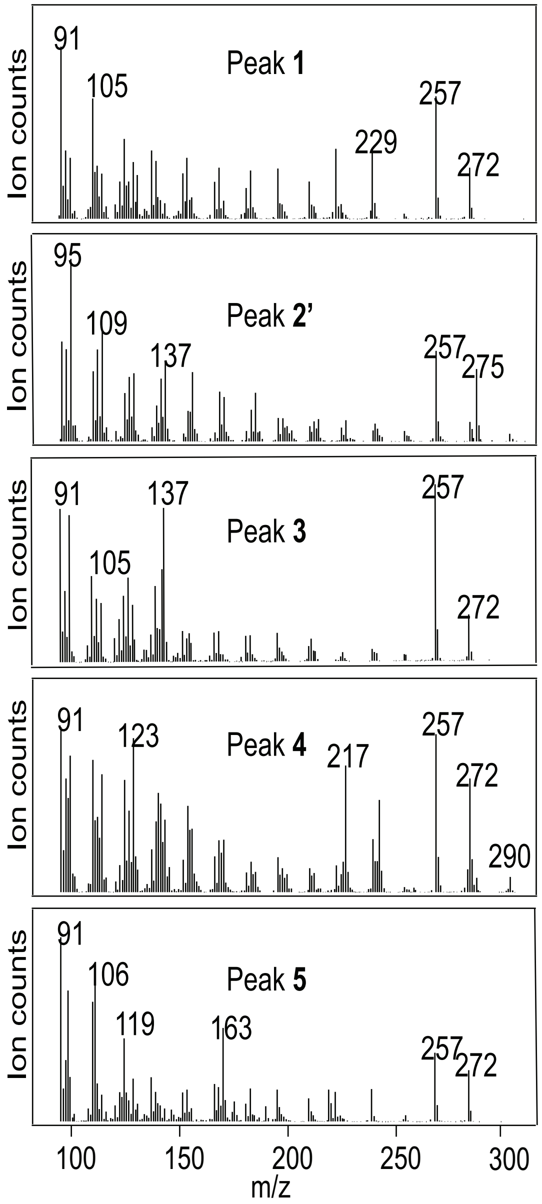

Figure S1. Mass spectra of the previously identified enzymatic product peaks from wild type and mutant KSs. Peak **1**: *ent*-kaurene; Peak **2'**: *ent*-copalol; Peak **3**: *ent*-pimara-8(14),15-diene; Peak **4**: 16α-hydroxy-*ent*-kaurane; Peak **5**: *ent*-isokaurene. Numbers correspond to the compound numbering defined in the text.

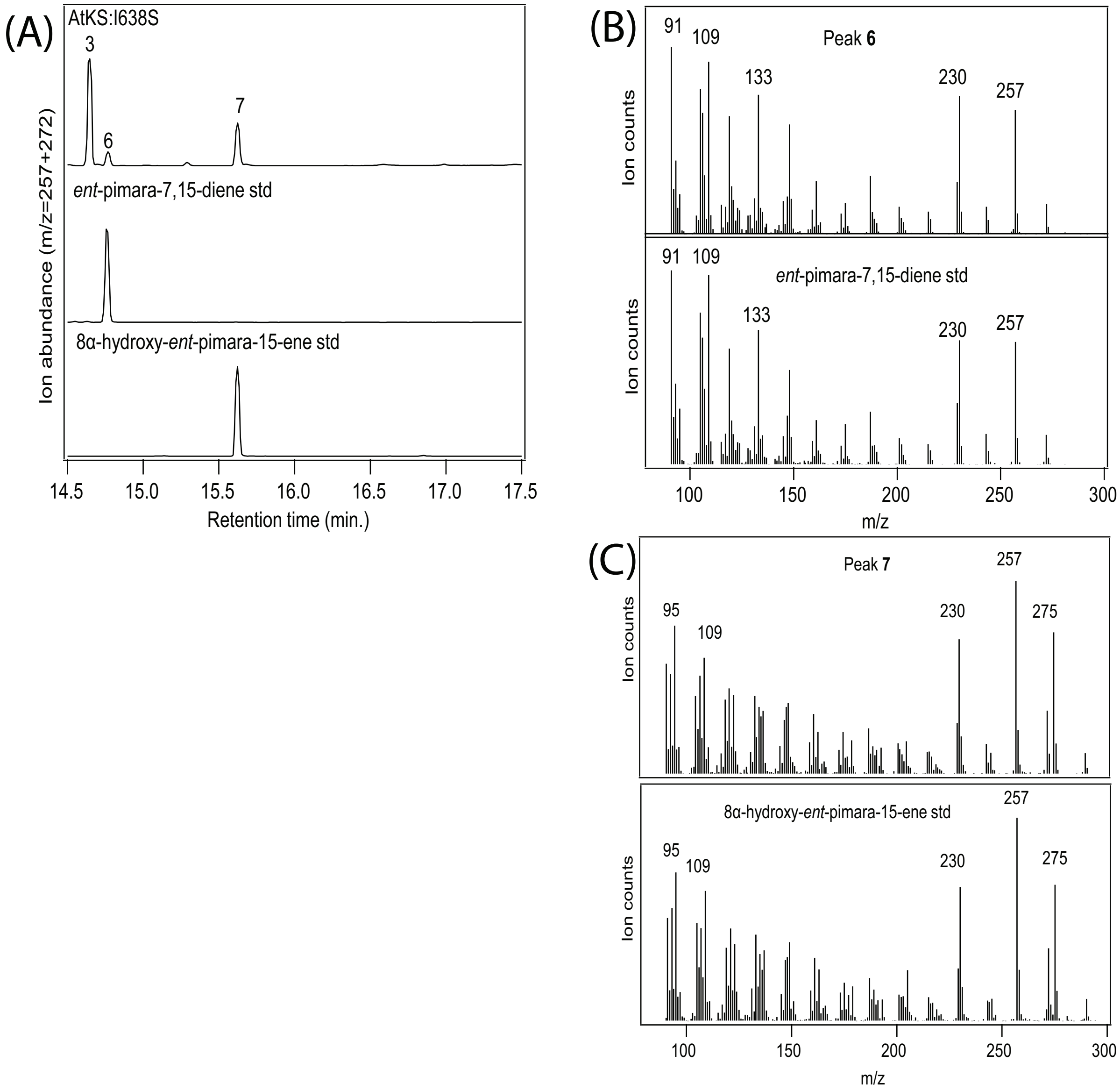

Figure S2. New enzymatic products of AtKS:I638S were identified by comparison of both retention time and mass spectrum to authentic standards. (A) Chromatograms from GC-MS analysis of the AtKS:I638S mutant and authentic standards. (B) Mass spectrum of the AtKS:I638S product peak **6** and mass spectrum of *ent*-pimara-8(14),15-diene standard (std). (C) Mass spectrum of the AtKS:I638S product peak **7** and mass spectra of 8α-hydroxy-*ent*-pimar-15-ene std. Numbers correspond to the compound numbering defined in the text.

Table S1: Primers for mutagenesis

Primer name	Sequence (with mutated position underlined)
MpKS:I645T-F	TTTGCACTTGGACCCACCGTTCCTGTAACAACTTTC
MpKS:I645T-R	GAAAGTTGTTACAGGAACGGTGGGTCCAAGTGCAAA
PpCPS/KS:I741T-F	TCCGTCGCACTAGAGCCCACCGTATGTAGCACTCTC
PpCPS/KS:I741T-R	GAGAGTGCTACATACGGTGGGCTCTAGTGCGACGGA
OsKSL5i:I664A-F	TTTGCTGTAGGCCCCGCGATCACCTCGGCAGCATTG
OsKSL5i:I664A-R	CAATGCTGCCGAGGTGATCGCGGGGCCTACAGCAAA
OsKSL5i:I664S-F	TTTGCTGTAGGCCCCAGCATCACCTCGGCAGCATTG
OsKSL5i:I664S-R	CAATGCTGCCGAGGTGATGCTGGGGCCTACAGCAAA
OsKSL5i:I664V-F	TTTGCTGTAGGCCCCGTGATCACCTCGGCAGCATTG
OsKSL5i:I664V-R	CAATGCTGCCGAGGTGATCACGGGGCCTACAGCAAA
AtKS:I638A-F	TTTGCATTAGGACCAGCGGTCCTCCCAGCTACCTAT
AtKS:I638A-R	ATAGGTAGCTGGGAGGACCGCTGGTCCTAATGCAAA
AtKS:I638S-F	TTTGCATTAGGACCAAGCGTCCTCCCAGCTACCTAT
AtKS:I638S-R	ATAGGTAGCTGGGAGGACGCTTGGTCCTAATGCAAA
AtKS:I638V-F	TTTGCATTAGGACCAGTGGTCCTCCCAGCTACCTAT
AtKS:I638V-R	ATAGGTAGCTGGGAGGACCACTGGTCCTAATGCAAA
